# Identification of SARS-CoV-2 Receptor Binding Inhibitors by In Vitro Screening of Drug Libraries

**DOI:** 10.3390/molecules26113213

**Published:** 2021-05-27

**Authors:** Alon Ben David, Eran Diamant, Eyal Dor, Ada Barnea, Niva Natan, Lilach Levin, Shira Chapman, Lilach Cherry Mimran, Eyal Epstein, Ran Zichel, Amram Torgeman

**Affiliations:** 1 Department of Biotechnology, Israel Institute for Biological Chemical and Environmental Sciences, Ness Ziona 7410001, Israel; alonb@iibr.gov.il (A.B.D.); erand@iibr.gov.il (E.D.); eyalo@iibr.gov.il (E.D.); adab@iibr.gov.il (A.B.); nivan@iibr.gov.il (N.N.); lilachl@iibr.gov.il (L.L.); lilachc@iibr.gov.il (L.C.M.); eyale@iibr.gov.il (E.E.); 2 Department of Pharmacology, Israel Institute for Biological, Chemical and Environmental Sciences, Ness Ziona 7410001, Israel; shirac@iibr.gov.il

**Keywords:** SARS-CoV-2, COVID-19, drug repurposing, spike protein, receptor binding domain (RBD), angiotensin-converting enzyme 2 (ACE2), high-throughput screening, small molecule inhibitors (SMIs)

## Abstract

Severe acute respiratory syndrome coronavirus 2 (SARS-CoV-2) is responsible for the coronavirus disease 2019 (COVID-19) global pandemic. The first step of viral infection is cell attachment, which is mediated by the binding of the SARS-CoV-2 receptor binding domain (RBD), part of the virus spike protein, to human angiotensin-converting enzyme 2 (ACE2). Therefore, drug repurposing to discover RBD-ACE2 binding inhibitors may provide a rapid and safe approach for COVID-19 therapy. Here, we describe the development of an in vitro RBD-ACE2 binding assay and its application to identify inhibitors of the interaction of the SARS-CoV-2 RBD to ACE2 by the high-throughput screening of two compound libraries (LOPAC^®^1280 and DiscoveryProbeTM). Three compounds, heparin sodium, aurintricarboxylic acid (ATA), and ellagic acid, were found to exert an effective binding inhibition, with IC50 values ranging from 0.6 to 5.5 µg/mL. A plaque reduction assay in Vero E6 cells infected with a SARS-CoV-2 surrogate virus confirmed the inhibition efficacy of heparin sodium and ATA. Molecular docking analysis located potential binding sites of these compounds in the RBD. In light of these findings, the screening system described herein can be applied to other drug libraries to discover potent SARS-CoV-2 inhibitors.

## 1. Introduction

Coronavirus disease 2019 (COVID-19) is a worldwide pandemic caused by severe acute respiratory syndrome coronavirus 2 (SARS-CoV-2). Currently, SARS-CoV-2 has infected over 150 million people, and over 3 million deaths have been reported [1]. The emergence of COVID-19 has prompted a global effort to develop vaccines and drugs for prevention and treatment. These efforts have led to the development of vaccines, several of which are being administered worldwide under emergency use authorization [2,3,4]. However, only a few therapeutics (Remdesivir and monoclonal antibodies) are available under emergency-use or conditional marketing authorization by the American and European regulatory authorities [5,6,7].

SARS-CoV-2 infects host cells via the binding of its spike glycoprotein (S) to human angiotensin-converting enzyme 2 (ACE2) on target cell surfaces [8,9]. The spike protein is composed of two functional subunits, S1 and S2. S1 includes the ACE2 binding domain (RBD), and the S2 subunit mediates the fusion of the virus to the infected cell membrane [9]. Due to its pivotal role in the virus infection process, the spike and, specifically, the RBD are major targets for generating vaccines and antibodies aiming to block the ACE2–spike interaction [2,3,4]. However, drug development, optimization, manufacturing, and preclinical and clinical studies are time consuming [10]. Alternatively, repurposing approved drugs for treating COVID-19 can potentially promote rapid and safe therapy [11,12].

An efficient strategy for drug repurposing is the high-throughput screening of drug libraries. Recent library screening studies have reported the discovery of several SARS-CoV-2 replication inhibitors [13] and compounds that target host proteins and exhibit antiviral activity by interfering with their interaction with viral proteins [14]. However, no compounds that can inhibit the RBD-ACE2 interaction have been found in these reports. Potential RBD-ACE2 inhibitors have been predicted in several in silico library screening studies only [15,16,17]. The addition of an in vitro screen of drug libraries can promote the discovery of potent inhibitors of the RBD-ACE2 interaction by gaining a functional layer of data.

The urgent need for a treatment prompted us to screen for approved drugs and well-known pharmaceutically active compounds to discover potential inhibitors of SARS-CoV-2 cell attachment in this study. To this end, an in vitro RBD-ACE2 binding assay was established and used to discover inhibitors of the interaction via the high-throughput screening of two compound libraries. One library, LOPAC^®^1280, contains 1280 pharmaceutically active compounds, and the other, DiscoveryProbe^TM^, contains 1363 FDA-approved drugs. We report herein the identification of three compounds (heparin sodium, aurintricarboxylic acid (ATA), and ellagic acid) that exhibit high inhibition activities. A plaque reduction assay in Vero E6 cells infected with a SARS-CoV-2 surrogate virus confirmed the inhibition efficacy of heparin sodium and ATA. A possible mechanism via molecular docking to the RBD is described.

## 2. Results

### 2.1. Experimental Design

**Figure 1:**
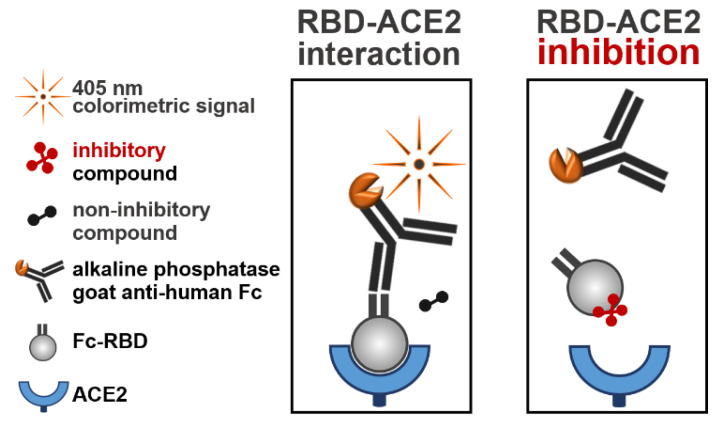
Schematic illustration of the RBD-ACE2 binding assay. Ninety-six-well plates were coated with ACE2 (100 ng/well) and blocked with TSTA buffer. Each compound (10 µM) from the libraries was incubated with Fc-RBD (1 µg/mL) for 1 h at 25 °C. Following incubation, mixtures were added to the ACE2-coated plates and incubated for 1 h at 37 °C. After washing, the plates were incubated for 1 h at 37 °C with the alkaline phosphatase-conjugated goat anti-human Fc fragment. The plates were then washed, and a colorimetric reaction was developed by the addition of *p*NPP (measured at 405 nm). Left panel: a maximum signal of an uninterrupted RBD-ACE2 interaction was obtained in the presence of noninhibitory compounds. Right panel: a reduced signal of an inhibited RBD-ACE2 interaction was obtained in the presence of inhibitory compounds.

The identification of compounds that inhibit the binding of RBD to ACE2 was carried out by high-throughput screening of the LOPAC^®^1280 and DiscoveryProbe^TM^ libraries using an assay designed to specifically measure the RBD-ACE2 interaction. This assay consisted of the SARS-CoV-2 receptor ACE2 adsorbed to 96-well plates and soluble hFc-RBD (Figure 1). The first step of the assay included preincubation of hFc-RBD with the tested compounds. The second step included incubation of the mixtures with the adsorbed ACE2. Finally, following a washing step, the RBD-ACE2 interaction was quantified by the addition of a detecting alkaline phosphatase-conjugated goat anti-human-Fc antibody. In the presence of a compound that could interfere with the RBD-ACE2 interaction, a lower signal was obtained. The hit compounds were further verified for their inhibitory effect by a plaque reduction assay conducted in Vero E6 cells with a surrogate virus (rVSV-SARS-CoV-2-S) expressing the SAR-CoV-2 spike protein.

### 2.2. Screening the LOPAC^®^1280 and DiscoveryProbe^TM^ Libraries for RBD-ACE2 Binding Inhibitors

**Figure 2:**
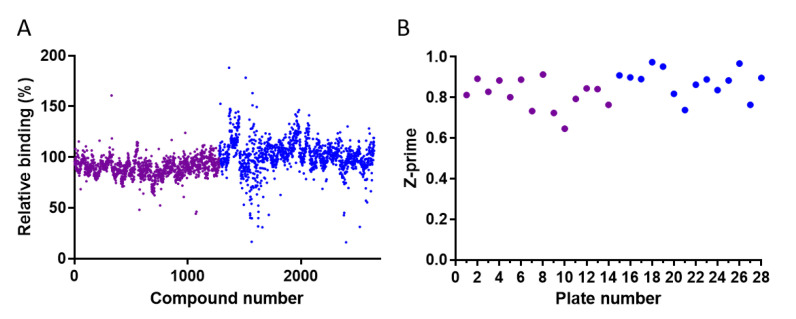
High-throughput screening of the LOPAC^®^1280 and DiscoveryProbe^TM^ compound libraries for inhibitors of the RBD-ACE2 interaction. Each compound was tested at 10 µM. (**A**) Distribution of the relative RBD-ACE2 binding values in the presence of the compounds. (**B**) Distribution of the Z-prime values of each plate. The purple and blue dots correspond to LOPAC^®^1280 and DiscoveryProbe^TM^, respectively.

The developed binding assay was used to screen two compound libraries for inhibitors of the RBD-ACE2 interaction. The relative binding in the presence of the tested compound (10 µM) was calculated by dividing the absorbance of the compound-containing well by that of the no-compound control wells (Figure 2A). The z-prime parameter of the screened plates, indicating the quality and power of the screening assay, was in the high acceptable range values (0.65–0.97) (Figure 2B).

**Figure 3:**
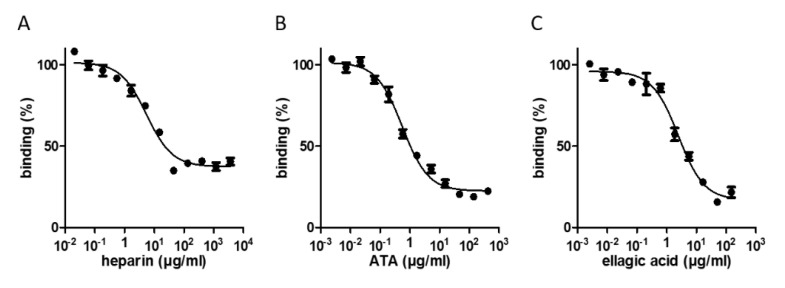
Dose–response relationships between the hit compound concentrations and RBD-ACE2 binding. Inhibition profiles of the RBD-ACE2 interaction in the presence of increasing concentrations of heparin sodium (**A**), ATA (**B**), and ellagic acid (**C**), determined by the binding assay. The values are the average of triplicates ± SEM. The IC_50_ values are listed in Table 1.

Applying an initial threshold of >40% inhibition (relative binding lower than 60%, Figure 2A) resulted in the selection of 30 and 22 compounds from the LOPAC^®^1280 and DiscoveryProbe^TM^ libraries, respectively. These hits were reassayed, and three molecules—heparin sodium, aurintricarboxylic acid (ATA), and ellagic acid—exhibiting a binding inhibition above 60% were further analyzed at different concentrations to determine their 50% inhibitory concentration (IC_50_) values. The three compounds displayed typical dose–response curves (Figure 3 and Table 1). ATA yielded the most potent IC_50_ value (0.6 µg/mL), followed by ellagic acid (2.5 µg/mL) and heparin sodium (5.5 µg/mL). The maximal binding inhibition of ATA and ellagic acid was ~80%, whereas heparin sodium exhibited a 63% inhibition.

### 2.3. Evaluation of the Selected Compounds by a Plaque Reduction Assay

**Figure 4:**
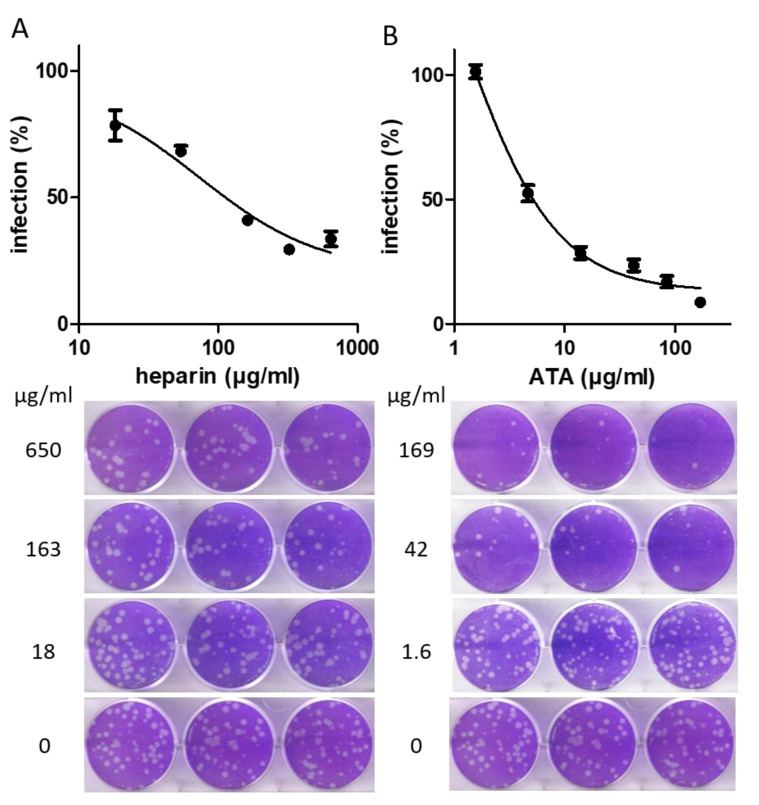
Inhibition of viral infection of cells by heparin sodium and ATA. rVSV-SARS-CoV-2-S was preincubated for 1 h with the indicated concentrations of heparin sodium (**A**) and ATA (**B**). Mixtures were then added to Vero E6 cells and incubated for 72 h. Cells were then stained using crystal violet, and the relative plaque reduction was determined. The results are expressed as the percent inhibition relative to that of the virus-only control incubated with the corresponding solvent (infection medium without or with 2% DMSO for heparin sodium or ATA, respectively). The values are presented as the mean of 6-well replicates ± SD. The lower images are three representative wells with the indicated compound concentrations.

The efficacy of the three identified molecules, heparin sodium, ATA, and ellagic acid, was further assessed by a plaque reduction assay conducted in Vero E6 cells using a SARS-CoV-2 surrogate virus (rVSV-SARS-CoV-2-S). The virus was preincubated with various concentrations of each molecule to ensure that potential interactions between the compounds and RBD would occur prior to RBD-ACE2 interaction. The mixtures were then added to cells in six-well plates for 72 h. The relative reduction in plaque formation was calculated, and IC_50_ was determined (Figure 4). ATA and heparin sodium displayed potent antiviral inhibition activities in cells (IC_50_ values of 5.6 and 73 µg/mL, respectively). For both compounds, the IC_50_ values determined using the plaque reduction assay were an order of magnitude higher than those determined using the binding assay, maintaining their relative ranking. A dose–response curve for ellagic acid could not be established, as concentrations in the range of 1–120 µg/mL of this compound did not inhibit rVSV-SARS-CoV-2-S infection in Vero E6 cells. These results demonstrate that ATA and heparin sodium are able to exert potent inhibition of viral infection, presumably by hindering the binding of the spike to cellular ACE2. To rule out a possible reduction in plaque formation due to cytotoxicity, the effect of the compounds at the highest tested concentrations on cell viability was evaluated using alamarBlue reagent, by comparing the viability of cells incubated with the compound to the viability of cells incubated only with media. None of the tested compounds were cytotoxic as the cell viability percentages following incubation with 120 µg/mL of ellagic acid, 169 µg/mL of ATA, and 650 µg/mL of heparin sodium were 106%, 102%, and 97%, respectively.

### 2.4. Molecular Docking Analysis

**Figure 5:**
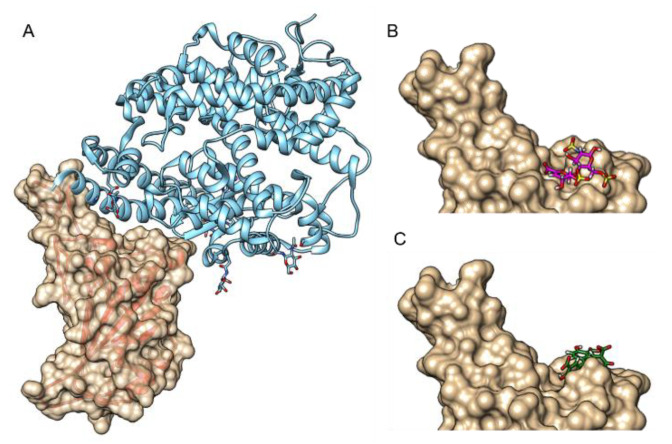
A docking model of the binding of heparin sodium and ATA to the RBD. (**A**) RBD-ACE2 interaction (pdb code 6M0J). The RBD forms a concave surface with a protruding loop on one side, to which ACE2 binds (ACE2 is colored in light blue and is shown in a ribbon presentation; RBD is shown in surface (gold colored) and ribbon (red colored) representations). Heparin sodium (one disaccharide unit) and ATA were docked to the RBM using the SwissDock server [20]. The compounds are shown in stick representation, where oxygen, sulfur, and hydrogen are colored in red, yellow, and white, respectively, and carbon atoms are colored in magenta for heparin sodium (**B**) and green for ATA (**C**). The figure was prepared using UCSF Chimera [21].

Molecular docking was conducted to suggest a mechanism underlying the inhibition of the RBD-ACE2 interaction by ATA and heparin sodium (Figure 5). Molecular docking was based on the published crystal structure of the RBD-ACE2 complex (resolution of 2.45 Å) [18]. The compounds were allowed to freely dock to the receptor binding motif (RBM)—the ACE2 binding site within the RBD [19]. The RBM spans amino acids 437 to 507 and forms a concave surface that interacts with ACE2. The compounds were docked to the concave surface of the RBM, implying that their binding to the RBD may disrupt the interaction with ACE2. The calculated Gibbs free energy (ΔG) values for the interaction of the compounds with the RBD were −8.9 and −7.05 kCal/mL for heparin sodium and ATA, respectively. The predicted binding site for heparin included Tyr505, which participates in the interaction of the RBD with ACE2. The predicted binding site for ATA was at a more pivotal location as, in addition to Tyr505, it includes Tyr449, Tyr453, Gln493, Gln498, and Asn501, found at the RBD-ACE2 interface.

## 3. Discussion

The global COVID-19 pandemic led to an urgent demand for developing vaccines and effective treatments. In view of this need, drug repurposing is a rapid approach for discovering beneficial COVID-19 therapeutics by using established drugs. Inhibiting the RBD-ACE2 interaction, thereby preventing viral attachment to target cells, is used as an approach to develop vaccines and therapeutics. The latter include antibodies, aptamers, peptides, and specific small-molecule inhibitors (SMIs) [22,23,24,25,26,27,28]. One effective and rapid strategy for discovering inhibitors is by screening compound libraries, as was recently applied to find new therapeutics for COVID-19. For example, Gordon et al. identified 332 new SARS-CoV-2-human protein–protein interactions and found two sets of pharmacological agents that displayed antiviral activity [14]. In another study, the ReFRAME (repurposing, focused rescue, and accelerated medchem) drug library was screened for inhibitors of viral replication, and 100 molecules, including 21 known drugs, were found [13]. However, no compound was associated with blocking the RBD-ACE2 interaction in either report. Screening for inhibitors, particularly SMIs, that can potentially hamper RBD-ACE2 binding has been reported in several in silico studies. Choudhary et al. identified five potential SARS-CoV-2 cell entry inhibitors that were shown to virtually bind the spike protein [15]. Wu et al. analyzed therapeutic targets in SARS-CoV-2 and virtually screened several compound databases for the inhibition of these targets. One compound (hesperidin) was suggested to bind the binding interface between the spike and ACE2 [17]. In the study of Wang et al., eight common types of potential inhibitor structures were outlined by applying a computational screening strategy based on blocking S RBD/hACE2 binding [16]. Despite the advantages of the in silico screening approach, the in vitro screening of drug libraries can add a functional level to the process, further promoting the discovery of potent inhibitors of the RBD-ACE2 interaction.

In the current study, a new binding assay was developed and used to screen two drug libraries for inhibitors that interfere with the RBD-ACE2 interaction. Three compounds that inhibited over 60% of the interaction were selected. Two compounds, heparin sodium and ellagic acid, are FDA-approved drugs. In addition to its anticoagulant activity, heparin has previously been found to inhibit infection by several viruses, including human immunodeficiency virus (HIV), coronavirus NL63, and Zika virus [29,30,31]. In line with our screening results, it has recently been shown that the spike protein of SARS-CoV-2 strongly binds immobilized heparin [32]. A docking analysis suggested putative heparin (and other glycosaminoglycans) binding motifs in the RBD when it is in an open conformation [32]. Interestingly, Clausen et al. [33] provided evidence that heparan sulfate, a cell membrane glycosaminoglycan, shifts the RBD to an open conformation to facilitate ACE2 binding. Additionally, it was suggested in their work that heparin could inhibit the role of heparan sulfate as a coreceptor for SARS-CoV-2 infection [33]. Most recently, heparin was found to inhibit SARS-CoV-2 infection in Vero cells [34]. The fact that heparin was specifically identified by the screening assay as a potent inhibitor strongly supports the reliability of the assay developed and applied herein for discovering effective compounds capable of directly blocking the RBD-ACE2 interaction. It is important to note that in an unrelated manner to SARS-CoV-2 inhibition, heparin is administered as an anticoagulant to COVID-19 patients suffering from coagulopathy and thromboembolism [35]. Thus, it appears that heparin is able to exert a beneficial synergistic effect in treating COVID-19 patients based on two distinct mechanisms. The third inhibiting compound is ATA, a polyanionic aromatic compound, which is recognized as a potent DNA topoisomerase II inhibitor [36]. Additionally, ATA exhibits inhibitory properties against several viruses and bacteria. Interestingly, among the viruses is the SARS coronavirus [37]. Other pathogens include HIV-1 [38], influenza virus [39], Vaccinia [40], Zika virus [41], and *Yersinia pestis* [42]. Ellagic acid, a polyphenol, is an investigational drug used to treat cancer, cardiovascular disease, and brain injury [43,44,45,46,47,48,49,50,51,52,53,54]. Similar to heparin and ATA, ellagic acid is also associated with antiviral activity against various viruses [55,56,57]. Recently, ellagic acid was identified by network analysis and molecular docking as a possible modulator of casein kinase II subunit alpha, a host protein that may be involved in the pathology of SARS-CoV-2 [58].

The three hit compounds were further characterized for their ability to inhibit the infection of Vero E6 cells by a pseudotyped virus expressing the SARS-CoV-2 spike protein using a plaque reduction assay. Only ATA and heparin sodium preserved their inhibitory activity in the cellular system. The IC_50_ values obtained in the cellular assay were an order of magnitude higher than those obtained in the binding assay, and in both assays, ATA demonstrated a higher inhibitory potency. Of note, the IC_50_ value obtained herein for heparin sodium is in line with that described in a recent report for the inhibition of the SARS-CoV-2 virus [34].

The ranking of IC_50_ values of ATA and heparin coincides with the results of the docking analysis. The higher potency of ATA can be explained in light of the prediction that ATA binds multiple residues of RBD at the interface of the RBD-ACE2 interaction, whereas heparin binds only one residue. The docking analysis may also serve to predict the binding of the identified compounds to SARS-CoV-2 variants. Of note, it has been reported that Asn501 is a critical residue for ACE2 binding by the RBM of SARS-like viruses [19]. This residue, according to the docking model, is part of the binding site of ATA. In this regard, several SARS-CoV-2 variants of concern listed by the CDC [59] (B.1.1.7, B.1.351, and P.1) contain an Asn501Tyr mutation. Although it can be hypothesized that a mutation in Asn501 might alter the binding of ATA to the RBD, the impact of the Asn501Tyr mutation on the potency of ATA is yet unknown. In the case of heparin, the docking analysis predicted that the binding site does not include residues mutated in the SARS-CoV-2 variants of concern. This suggests that the heparin binding to RBD of SARS-CoV-2 variants may not be altered.

No effective inhibition of the rVSV-SARS-CoV-2-S virus could be demonstrated in Vero E6 cells by ellagic acid. It should be noted that the elimination of hit compounds between the primary and secondary screens is a characteristic of the screening funnel. However, we cannot rule out the possibility that, although ineffective in cells, ellagic acid could still be potent in vivo. Eubanks et al., for example, reported that two botulinum toxin inhibitors were effective in vivo even though they were ineffective in a cellular assay [60]. Further investigation is required to determine the inhibitory activity of ellagic acid against SARS-CoV-2.

In the absence of a specific anti-SARS-CoV-2 therapy, repurposing approved drugs may be useful for treating COVID-19 [11,61]. The current study can expand the limited drug toolkit by identifying approved drugs and pharmaceutically active compounds that can potentially be repurposed to inhibit the viral cell attachment step. The results provide a basis for further research with SARS-CoV-2 to evaluate the efficacy of hit compounds. Future structure–activity research (SAR) may allow one to design modifications that enhance the affinity of the compounds, required to achieve improved therapeutic efficacy and selectivity. Interestingly, as these drugs have been reported to counteract additional viruses as well, they may serve as broad-spectrum antiviral therapeutics. These drugs can also be considered for use in a combined administration to increase the treatment efficacy, which may be further advantageous as their doses in a combined regimen can be reduced, thus minimizing the risk of side effects. Moreover, the binding assay developed herein can be applied for the high-throughput screening of additional compound libraries to facilitate the discovery of additional potent compounds.

## 4. Materials and Methods

### 4.1. Expression and Purification of ACE2 and Fc-RBD

A synthetic gene encoding the peptidase domain of *Homo sapiens* ACE2 (amino acids 19-615, GenBank: BAB40370.1), with optimized codon usage for expression in *Escherichia coli* and a C-terminal His-tag, was prepared by GenScript (Piscataway, NJ, USA) and cloned into pET-9a between the NdeI and BamHI sites. The plasmid pET-9a-ace2 was transformed into *E. coli* BL21(DE3). To express the protein, *E. coli* BL21(DE3)+pET-9a-ace2 was grown at 18 °C in TB media and was harvested after 40 h of incubation. To purify the protein, the cell pellet was resuspended in binding buffer (20 mM of sodium phosphate, 0.5 M of NaCl, and 20 mM of imidazole; pH 7.4; 100 mL per 25 g of wet cells) and disrupted by three passages in a cell homogenizer at 25 kPa (C series cell disruptor 1.1 KW, Constant Systems). The cell extract was centrifuged (14,000 g, 30 min) and the supernatant was loaded onto a 5 mL HisTrap FF column (GE Healthcare, Chicago, IL, USA) mounted on an AKTA Explorer FPLC system (GE Healthcare). The column was washed with 10 CV of binding buffer and 10 CV of binding buffer containing 40 mM of imidazole. The protein was eluted from the column with elution buffer (20 mM of sodium phosphate, 0.5 M of NaCl, 500 mM of Imidazole, pH 7.4). The purified protein was dialyzed against PBS and stored at −70 °C. The recombinant hFc-RBD fusion protein, expressed as recently described [22], was kindly provided by Dr. Ohad Mazor Lab (IIBR, Ness Ziona, Israel).

### 4.2. RBD-ACE2 Binding Assay

Ninety-six-well plates (Maxisorp, Nunclon) were coated with ACE2 (100 ng/well) for 1 h at 37 °C. The plates were blocked for 1 h at 37 °C with TSTA buffer (2% (*w*/*v*) bovine serum albumin (BSA), 0.9% NaCl, 0.05% Tween 20, 50 mM of Tris, pH 7.6). For initial screening, a concentration of 10 µM of each compound from the LOPAC^®^1280 (Sigma, St. Louis, MO, USA) and DiscoveryProbe^TM^ FDA-approved drug (APExBIO) libraries was incubated with 1 µg/mL of Fc-RBD in a total reaction mixture volume of 50 µL. For the IC_50_ experiments, 3-fold serial dilutions of the indicated compound (1 mM to 5.6 nM) were incubated with Fc-RBD (1 µg/mL). The mixtures (50 µL) were incubated for 1 h at 25 °C. Following incubation, the mixtures were added to the ACE2-coated plates and incubated for 1 h at 37 °C. After washing, the plates were incubated for 1 h at 37 °C with that alkaline phosphatase-conjugated goat anti-human Fc fragment (Sigma, St. Louis, MO, USA), diluted 1:1000 in blocking solution. The plates were washed, and the colorimetric reaction was developed using *p*-nitrophenyl phosphate (*p*NPP, Sigma, St. Louis, MO, USA). The absorbance was measured at 405 nm with a Synergy HTX ELISA reader (Biotek, USA). The assay included a rabbit anti-SARS-CoV-2 RBD polyclonal antibody as a positive control. The antibody was kindly provided by Dr. Ohad Mazor Lab (IIBR, Ness Ziona, Israel). Naïve rabbit serum served as a negative control. The IC_50_ values were calculated using a 4-parameter logistic fit by Gen5 software (Biotek, Winooski, VT, USA). The z-prime parameter for each plate was calculated by Gen5 software.

### 4.3. Plaque Reduction Assay

The assay was conducted in Vero E6 cell monolayers (ATCC^®^ CRL-1586™). The cells were grown in growth medium (Dulbecco’s modified Eagle’s medium (DMEM) containing 10% fetal bovine serum (FBS), 1% MEM nonessential amino acids (NEAA), 2 mM of L-glutamine, and 0.5% Pen/Strep, all from Biological Industries, Kibbutz Beit-Haemek, Israel) and were cultured at 37 °C and 5% CO_2_. The cells were infected with 60 PFU/well of rVSV-SARS-CoV-2-S, which served as a SARS-CoV-2 surrogate virus. This virus is a replication-competent recombinant VSV-ΔG-spike virus in which the glycoprotein of VSV (vesicular stomatitis virus) is replaced by the spike protein of SARS-CoV-2 [62]. Cells were seeded in 6-well plates (7 × 10^5^ cells/well) and grown overnight in growth medium. A fixed dose (600 PFU/mL) of rVSV-SARS-CoV-2-S was incubated with different inhibitor concentrations of ATA, ellagic acid, and heparin (1:1, *v*/*v*) in infection medium (MEM containing 2% FBS with 1% NEAA, 2 mM of glutamine, and 0.5% P/S) and was used to infect Vero E6 monolayers in six replicates (200 µL/well). After incubation for 1 h at 37 °C, 3 mL/well of overlay (MEM containing 2% FBS and 0.4% tragacanth (Merck, Herzliya Pituach, Israel)) was added to each well, and the plates were incubated at 37 °C and 5% CO_2_ for 72 h. The media were then aspirated, and the cells were fixed and stained with 3 mL/well of crystal violet solution (Biological Industries, Kibbutz Beit-Haemek, Israel). The number of plaques in each well was determined, and the reduction in infection was calculated relative to the control wells containing no compound. The IC_50_ values were calculated using GraphPad Prism.

### 4.4. Cytotoxicity assaY

A cytotoxicity assay was performed in Vero E6 cell monolayers using the alamarBlue cell viability reagent (Thermo Fisher Scientific). Cells (2 × 10^4^ cells/well in 100 μL) were seeded in 96-well plates. After an overnight incubation at 37 °C, 80 μL of medium was aspirated, and 20 μL of inhibitors was added in triplicate to the wells. After 1 h of incubation, 20 μL was aspirated, 150 μL of overlay was added, and the cells were incubated at 37 °C for three days. Then, 19 μL of alamarBlue reagent was added per well and incubated for 2.5 h at 37 °C. The fluorescence results were determined by excitation at 530 nm and the collection of emission at 580 nm was determined using a CLARIOstar^®^ reader (BMG labtech, Ortenberg, Germany). The cell viability was calculated by dividing the fluorescence results of the compound-containing wells by those of the control wells (containing no compounds).

### 4.5. Molecular Docking Analysis

Molecular docking of the selected compounds was performed using the SwissDock server (www.swissdock.ch accessed on 7 October 2020 [20]). The RBD of pdb file 6M0J [18] was set as a target, and the 3D structures of the hits were from the ZINC database [63].

## Figures and Tables

**Table 1:** RBD-ACE2 inhibition properties of the hit compounds.

Compound	Structure	IC_50_ [µg/mL]	Binding Inhibition [%]
heparin sodium	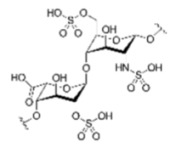	5.5	63
aurintricarboxylic acid (ATA)	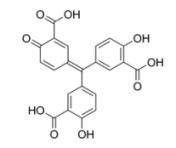	0.6	80
ellagic acid	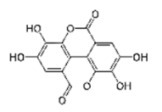	2.5	84

## Data Availability

The data presented in this study are available on request from the corresponding authors.

## Sample Availability

Samples of the compounds are available from the authors.
